# Retinal Vascular Pathology in a Rat Model of Cerebral Small Vessel Disease

**DOI:** 10.3389/fneur.2020.00533

**Published:** 2020-06-30

**Authors:** Heinrich Maximilian Scheifele, Philipp Ulbrich, Cornelia Garz, Roxana Octavia Carare, Hans-Jochen Heinze, Stefanie Schreiber, Solveig Jandke

**Affiliations:** ^1^Department of Neurology, Otto-von-Guericke University, Magdeburg, Germany; ^2^German Center for Neurodegenerative Diseases (DZNE) Within the Helmholtz Association, Magdeburg, Germany; ^3^Department of Behavioral Neurology, Leibniz Institute for Neurobiology (LIN), Magdeburg, Germany; ^4^Faculty of Medicine, University of Southampton, Southampton, United Kingdom

**Keywords:** small vessel disease (SVD), retina, spontaneously hypertensive stroke-prone rat (SHRSP), cerebral amyloid angiopathy (CAA), hypertensive arteriopathy (HA)

## Abstract

**Introduction:** The initial disease stages of hypertensive arteriopathy (HA) and cerebral amyloid angiopathy (CAA), the two main forms of sporadic human cerebral small vessel diseases (CSVD), are too subtle to be detectable on clinical routine imaging. Small vessel disease (SVD) is a systemic condition, affecting not only the brain, but also other organs. The retina appears as an ideal marker for the early detection of incipient CSVD. We therefore investigated the retinal microvasculature of the spontaneously hypertensive stroke-prone rat (SHRSP), an animal model of sporadic CSVD.

**Materials and Methods:** The brains and retinas of 26 male SHRSP (18–44 weeks) were examined histologically and immunohistochemically for the presence of HA phenomena (erythrocyte thrombi, small perivascular bleeds) and amyloid angiopathy (AA).

**Results:** CAA and AA in the retina showed a significant correlation with age (CAA: rho = 0.55, *p* = 0.005; AA: rho = 0.89, *p* < 0.001). The number of erythrocyte thrombi in the brain correlated with the severity of retinal erythrocyte thrombi (rho = 0.46, *p* = 0.023), while the occurrence of CAA correlated with the appearance of AA in the retina (rho = 0.51, *p* = 0.012). Retinal SVD markers predicted CSVD markers with good sensitivity.

**Conclusions:** These results indicate that SVD also occurs in the retinal microvasculature of SHRSP and the prediction of cerebral erythrocyte thrombi and CAA might be possible using retinal biomarkers. This underlines the important role of the investigation of the retina in the early diagnosis of CSVD.

## Introduction

Sporadic cerebral small vessel disease (CSVD) describes clinical, cognitive, neuroradiological, and histological findings based on pathological alterations of the cerebral microvasculature (1, 2). It is a very common disease in the aging population, found in the brains of up to 76% of non-demented individuals (3). Hypertensive arteriopathy (HA) and cerebral amyloid angiopathy (CAA) resemble the two main forms of sporadic human CSVD (1, 4); HA is defined by an early small vessel wall damage (2, 5) whereas CAA is characterized by the accumulation of β-amyloid (Aβ) in the walls of arteries and capillaries (6, 7). CSVD accounts for about 20–25% of ischemic strokes worldwide and for about 50% of intracerebral hemorrhages (8–10). There is, however, a presumably long pre-symptomatic clinically silent stage, during which CSVD dynamically evolves but can still be halted or delayed by the control of vascular risk factors (10).

During that clinically silent period, HA and CAA are already detectable *in vivo* applying the STandards for ReportIng Vascular changes on nEuroimaging (STRIVE) or the modified Boston criteria with magnetic resonance imaging (MRI) (2, 11). The imaging criteria include CSVD downstream pathologies, such as white matter hyperintensities (WMH) or lacunes, indicating already proceeding small vessel disease (12).

However, CSVD is initiated much earlier by direct vascular damage (for HA), and by the deposition of Aβ within the basement membranes of the smooth muscle cells (for CAA) (13). At this time, downstream pathologies are infrequent, deeming those initial disease stages too subtle to be detectable on clinical routine MRI (10). There is thus a need for further markers indicating the initial stages of the disease process, which should be potentially highly susceptible for disease-modifying strategies (14, 15).

Small vessel disease (SVD) is a systemic condition, affecting not only the brain, but also other heavily perfused organs (16–19). There are, however, no studies available that relate retinal SVD and more initial or direct CSVD pathologies to one another.

The retina appears an ideal biomarker for the early detection of incipient CSVD: it has a strong anatomical and functional similarity as well as a high spatial proximity to the brain. It can prospectively serve as an *in vivo* marker in clinical trials allowing for repeated monitoring of disease progression and modification (20–22). In that instance, retinal vessel alterations (e.g., arteriolar narrowing, arteriovenous nickening, microbleeds, or microaneurysms) have been demonstrated to parallel CSVD development and correlate with the severity of HA- and CAA-downstream-MRI markers, i.e., WMH and lacunes (17–19, 23, 24). However, there is no data on the relationship of initial retinal SVD to potentially use it as a biomarker for initial microvascular brain disease. We thus here aimed to investigate the retinal microvasculature in an experimental model of CSVD to relate early retinal and brain SVD markers to one another.

Due to the combination of a vascular risk profile with genetically anchored arterial hypertension, spontaneous infarct development and typical microangiopathic histology, we have chosen the spontaneously hypertensive stroke-prone rat (SHRSP) as an experimental CSVD model (25, 26). Male SHRSP show an age-dependent, progressive arterial hypertension with initial systolic blood pressure values of around 140 mmHg at 5 weeks of age raising to around 220 mmHg at 20 weeks of age (27, 28). Histopathological findings in the SHRSP include degenerative wall changes in small vessels with accumulation of plasma proteins, blood-brain barrier (BBB) breakdown, non-occlusive and occlusive thromboses and infarcts in the basal ganglia and cortical regions (29–31). Initial CSVD, in terms of endothelial disturbances and BBB breakdown can be already detected at an age between 12 and 18 weeks; while later disease stages, i.e., occlusive thromboses are usually found not before an age of 28–32 weeks (29, 31–33). Our previous work has also depicted vascular amyloid accumulations, in terms of CAA, in SHRSP which became obvious first at ages around 18–20 weeks but more prominent from 24 weeks on (32, 34).

## Materials and Methods

### Animals

The approval for animal testing was obtained from the Animal Care Committee of Saxony-Anhalt (reference numbers: 42502-2-1148 DZNE and 42502-2-1277 Uni/MD). In total, 26 male SHRSP (Charles River Laboratories International Inc., Wilmington, MA, USA) aged from 18 to 44 weeks were investigated (18 weeks: *n* = 5, 24 weeks: *n* = 5, 28 weeks: *n* = 5, 32 weeks: *n* = 6, 44 weeks: *n* = 5). Based on our previous research these age groups picture initial as well as advanced small vessel pathologies, although the onset of the disease can vary. All animals were housed under standard conditions (room temperature, 12 h light/dark cycle, free access to food and water). The health status was monitored by a daily assessment of neurological functions (such as decreased spontaneous activity, coordination failure, falling to one side, and hunched posture) and supported by weekly weight controls. All animals were neurologically inconspicuous during the entire observation period.

### Histology

#### Tissue Preparation

For anesthesia, Pentobarbital (40 mg/kg body weight) was injected intraperitoneally in all animals. Transcardial perfusion was conducted with 120 mL of phosphate buffered saline (PBS) followed by fixation with 120 mL of 4% paraformaldehyde (PFA) within 8 min. Brains and eyes were removed and stored for 48 h (brains in 4% PFA, eyes in 3.7% formalin). For cryoprotection, the organs were stored in 30% sucrose for 6 days and then frozen in methylbutane at −80°C. Twenty-four hours before cutting, the organs were stored at −20°C. Tissue sections (30 μm) of all brains and the right eye were prepared using a cryostat.

##### Brain

For all animals, the brains were sliced from the frontal to the occipital pole and coronal slices of 10–11 sectional planes were taken. Five adjacent slices per plane (in total 50 or 55 slices per animal) covering all brain regions (cortex, basal ganglia, hippocampus, corpus callosum, thalamus) oriented to “The rat brain in stereotaxic coordinates” (35) were used for histological staining. The first three slices were used for hematoxylin and eosin (HE) staining in order to identify early HA phenomena (see below). The following two slices were used for Congo red (CR) and Thioflavin T (ThioT) staining to detect vascular Aβ deposits in terms of CAA.

For HE staining the brain slices were washed twice with distilled water, incubated with hematoxylin (Carl Roth GmbH + Co. KG, Karlsruhe, GER), washed again, followed by bluing under running tap water and another distilled water rinse. Afterwards, staining was performed with 1% eosin solution (Carl Roth GmbH + Co. KG, Karlsruhe, GER) for 40 s. For CR and ThioT staining, the slices were washed, stained with nuclear fast red (Sigma-Aldrich Co. LLC, Steinheim, GER) for 5 min to label nuclei, rinsed with tap water for 10 min and incubated for 30 min in CR solution (Carl Roth GmbH + Co. KG, Karlsruhe, GER) or for 8 min in 1% ThioT solution (Sigma-Aldrich Co. LLC, Steinheim, GER), respectively. Dehydration and fixation were performed similarly in all stainings. After dehydration with increasing concentrations of Rotisol (Carl Roth GmbH + Co. KG, Karlsruhe, GER) the tissue was embedded in Xylene (Carl Roth GmbH + Co. KG, Karlsruhe, GER) and mounted with coverslips using histomount (Fisher Scientific GmbH, Schwerte, GER).

##### Retina

Coronal slices of the entire eye were prepared from the frontal to the distal pole at the exit of the optic nerve. Ten adjacent slices from four sectional planes (40 slices per animal) were used for histological stainings. In each plane the first four slices were used for HE staining (used for the detection of the same HA phenomena seen in the brain; see below) and the following six slices were used for CR and ThioT staining [aimed for the detection of amyloid angiopathy (AA)]. Blood vessels in all retinal layers as well as in the adjacent choroid (suprachoroidal layer and choriocapillaris) were investigated. Both vascular networks can thereby considered together as they both derive from the ophthalmic artery, are exposed to systemic vascular risk factors and are involved in supplying the retina with blood (36).

### Immunohistochemistry

Besides conventional histological Aβ detection, the occurrence of (peri)vascular Aβ and additionally the plasma protein Immunglobulin G (IgG) to detect early (C)SVD in terms of blood brain barrier (BBB) and blood retina barrier (BRB) breakdown, was examined immunohistochemically in *n* = 5 slices per brain and *n* = 12 slices per eye adjacent to the slices used for histological staining. Immunohistochemistry was performed in the brains of *n* = 24 SHRSP (mean age: 30.0 weeks) and in the eyes of *n* = 11 exemplary SHRSP (18 weeks: *n* = 2, 24 weeks: *n* = 3, 32 weeks: *n* = 3, 44 weeks: *n* = 3; mean age: 30.5 weeks). In short, tissue was pretreated with citrate buffer (70°C, 30 min), slices were repeatedly washed in PBS and blocked with 10% donkey serum. Subsequently, slices were stained with STL-FITC (solanum tuberosum lectin-fluorescein isothiocyanate, endothelial marker, 1:500; Axxora, Enzo Life Sciences GmbH, Lörrach, GER) and anti-rodent Aβ (1:500; Covance, Dedham, MA, USA; specific for rodent Aβ) overnight at 4°C. Slices were then incubated with Cy3-donkey anti rat IgG (1:200, BBB/BRB breakdown detection; Jackson Immuno Research, West Grove, PA, USA) and Cy5-donkey anti rabbit IgG (1:500, detection of Aβ; Jackson Immuno Research) for 2 h at room temperature. Finally, DAPI (4′,6-diamidino-2-phenylindol, nuclear staining, 1:10,000; MoBiTec GmbH, Göttingen, GER) staining was performed for 20 min at room temperature. After dehydration with increasing concentrations of alcohol, slices were mounted on slides with Histomount (Fisher Scientific GmbH, Schwerte, GER).

### Quantification

The following SVD phenomena were quantified in the brain and the retina: (i) non-occlusive erythrocyte thrombi defined as the accumulation of single or multiple erythrocytes in the lumen of the vessels (subsequently referred to as erythrocyte thrombi), (ii) small perivascular bleeds defined as leakage of erythrocytes out of the vessel, and (iii) the accumulation of Aβ in the small vessel wall.

To investigate the prevalence in our sample, the presence of a certain pathology was first assessed in a binary manner (i.e., existent, not existent). The number of affected vessels was further counted to, respectively, evaluate the severity of each pathology per animal. To prove that the number of affect vessels does not differ because of alterations in the vessel density, we also counted the number of capillaries and arterioles per FOV in all five brain regions of 6 exemplary animals (18 weeks: *n* = 2; 28 weeks: *n* = 2; 44 weeks: *n* = 2). In total, 15 FOVs in 3 HE stained brain slices of each animal were analyzed.

For both, brain and retina, a distinction was made between capillary and arteriolar vessels for quantifying erythrocyte thrombi. Vessels with a diameter from 20 to 65 μm were considered arterioles (37). Vascular connections between arterioles and venules with a diameter < 20 μm were considered capillaries (38).

For brain pathology, non-occlusive erythrocyte thrombi and small perivascular bleeds (HE staining) were counted in 50 randomly chosen fields of view (FOVs) per brain region within 10–11 sectional planes per animal (three slices from each sectional plane). For statistical analysis the mean of all FOVs per brain region was used. For CR and ThioT staining the entire slices (but still separated for each brain region) were investigated and amyloid positive vessels were summed up for each animal. For retinal pathology, the entire slices were investigated for HE, CR and ThioT staining.

Analysis of immunohistochemical staining took account of the entire slices for the brain and the retina, respectively. Assessment of perivascular/parenchymal IgG and vascular Aβ accumulation was performed in a binary manner (existent or not existent).

### Statistical Analysis

Statistical analysis was conducted in *n* = 24 SHRSP as two animals (*n* = 1, 18 weeks and *n* = 1, 24 weeks) were defined as outliers (number of cerebral erythrocyte thrombi exceeded the threshold of 2.5 standard deviations from the mean) and therefore excluded from the analysis.

Non-parametric Kruskal-Wallis test was used to compare erythrocyte thrombi, small perivascular bleeds and vascular Aβ accumulations between the 5 age groups (18, 24, 28, 32, 44 weeks). Non-parametric correlations were further conducted to relate erythrocyte thrombi, small perivascular bleeds and vascular Aβ accumulations to age. For the brain, these tests were conducted for the whole brain and separately for each single brain region (cortex, basal ganglia, hippocampus, corpus callosum, and thalamus).

To assess the association between the different SVD phenomena (erythrocyte thrombi, small perivascular bleeds, CAA/AA) in the brain and the retina non-parametric correlations were calculated with the brain considered as a whole and region-wise, respectively.

*P*-value ≤ 0.05 were deemed statistically significant. For *post-hoc* pairwise comparisons (10 tests for *n* = 5 age groups) as well as non-parametric correlation analysis between brain regions (5 tests for *n* = 5 regions) *p*-values were Bonferroni adjusted for multiple testing (corrected threshold for *post-hoc* pairwise comparison *p* ≤ 0.005, corrected threshold for brain regions *p* ≤ 0.01).

To test the predictive value of retinal SVD (erythrocyte thrombi, AA) for the severity of whole-brain CSVD (erythrocyte thrombi, small perivascular bleeds, CAA), Receiver-Operating-Characteristic (ROC) curve analyses were performed. Retinal small perivascular bleeds were excluded from this analysis; because of their overall low number in this sample (see Results) they were not powered enough to predict the severity of any cerebral pathology. SHRSP were dichotomized into “weakly” or “severely” affected animals: for cerebral erythrocyte thrombi median-split based threshold of 81 erythrocyte thrombi per 250 FOVs was chosen. For CAA, the number of Aβ-positive vessels increased with age (see Results). Therefore, a first ROC curve analysis was conducted, in which age was the state variable and the number of Aβ-containing vessels was the test variable. Based on the highest Youden-index a threshold of 30 Aβ-positive vessels in the whole brain was chosen. For cerebral small perivascular bleeds, prevalence (existent, not-existent) was taken for ROC curve analysis because of their overall low number in this sample.

The cutoff values of retinal pathologies with the highest combined sensitivity and specificity were determined by calculating Youden's J statistic.

For immunohistochemistry, differences of IgG and Aβ between whole-brain and retina for the whole sample were assessed by logistic regression analysis. *P*-value ≤ 0.05 were deemed statistical significant.

All statistical tests were performed applying “IBM SPSS Statistics 25” (IBM Corp., Armonk, NY, USA).

## Results

### Brain

**Figure 1:**
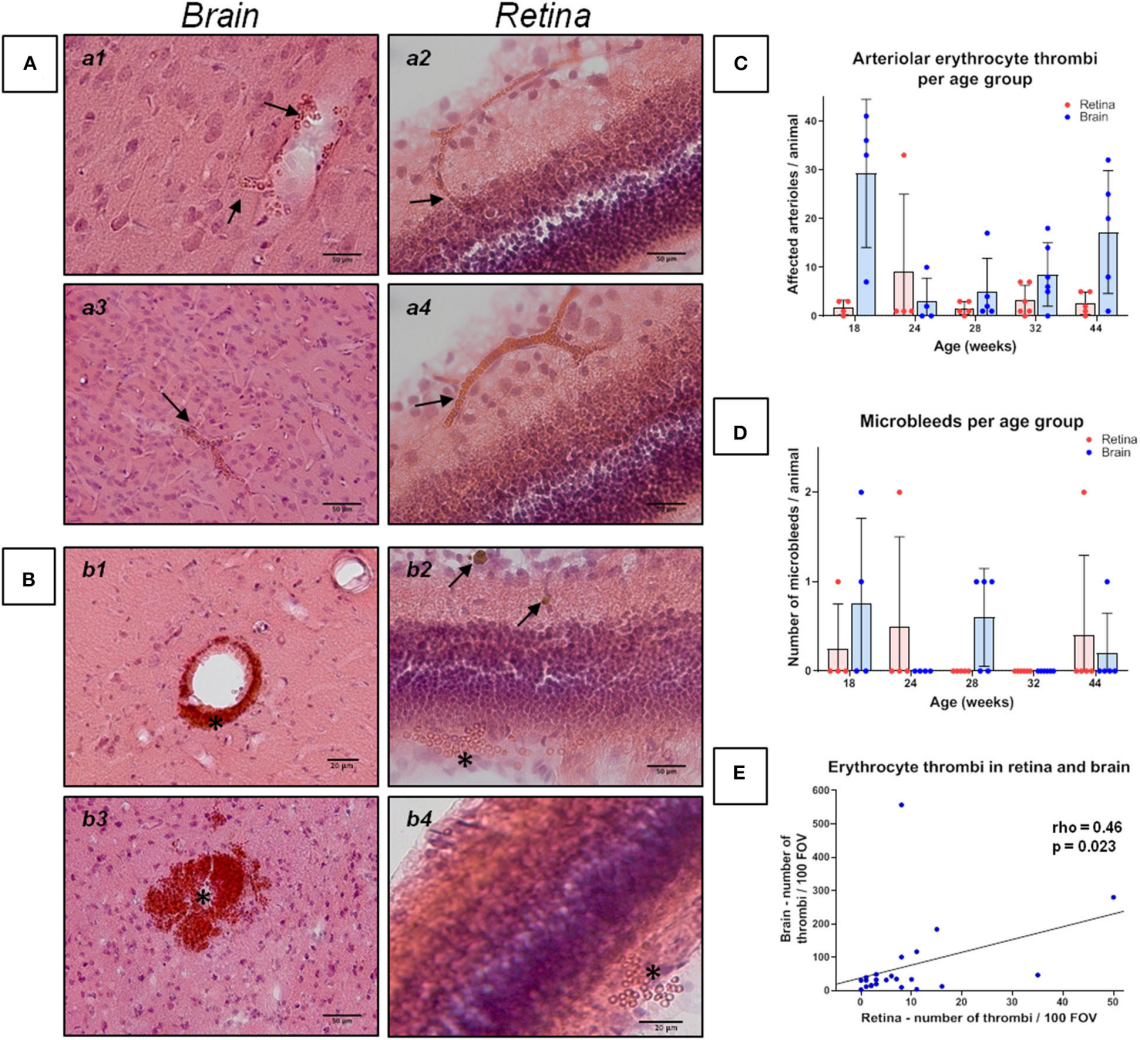
Erythrocyte thrombi and small perivascular bleeds in the brain and retina of spontaneously hypertensive stroke-prone rats. **(A,B)** The left panels (a1, a3, b1, b3) display small vessel disease (SVD) pathological features of the brain, whereas the right panel (a2, a4, b2, b4) display SVD of the retina. In all retina pictures, the inner retinal layers are shown at the top continuing to the outer layers or the choroid at the bottom. All pictures in **(A)** show cerebral and retinal erythrocyte thrombi (arrows), while pictures in **(B)** display small perivascular bleeds (asterisks). One small perivascular bleed in the retina (b2, asterisk) is accompanied by the occurrence of siderophages (b2, arrows) indicating an older bleed. **(C)** The diagram shows the retinal and cerebral mean number (±SEM) of arteriolar erythrocyte thrombi per animal across all age groups. Each dot represents the number of one investigated animal. There are no significant differences between the age groups. Surprisingly, and in contrast to former studies, 18 weeks old SHRSPs display the highest number of the shown pathology. **(D)** The diagram shows the retinal and cerebral average number (±SEM) of small perivascular bleeds per animal across all age groups. Each dot represents the data from one investigated animal. There were no significant differences between the age groups. **(E)** The scatterplot shows the significant correlation between the occurrence of erythrocyte thrombi in the retina and in the brain. Each dot represents the data from one investigated animal. The number of erythrocyte thrombi was normalized to 100 FOVs for retina and brain, respectively. **(A,B)** Hematoxylin-Eosin-staining.

**Figure 2:**
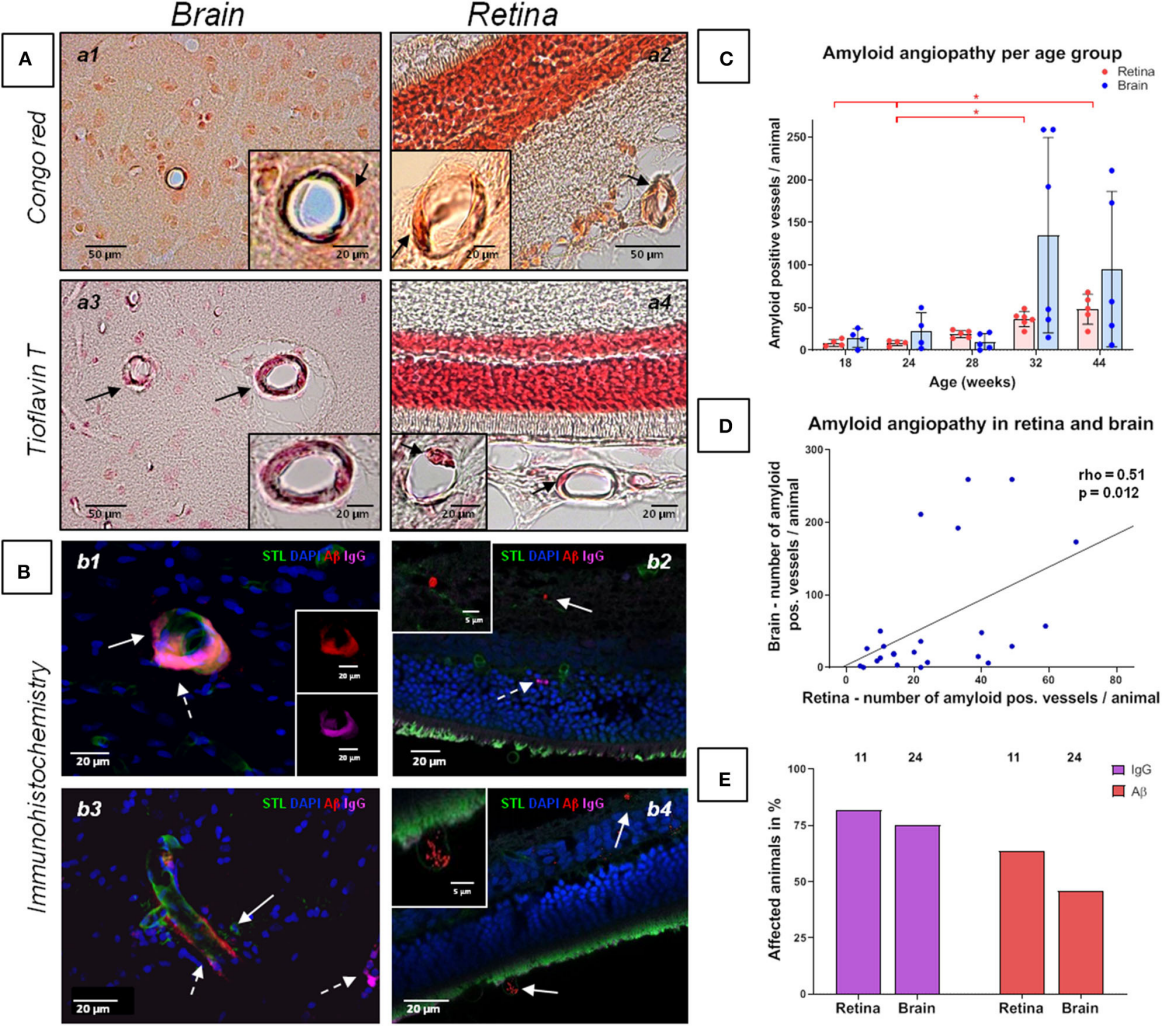
Amyloid angiopathy of the cerebral, retinal and choroidal vasculature in spontaneously hypertensive stroke-prone rats. **(A,B)** The left panels (a1, a3, b1, b3) display amyloid angiopathy of the brain (arrows), whereas the right panel (a2, a4, b2, b4) display amyloid angiopathy in the retina and the choroid (arrows). Additionally to Aβ deposits, IgG positive vessels could be detected (b2, dashed arrow). In all retina pictures, the inner retinal layers are shown at the top continuing to the outer layers or the choroid at the bottom. The dark orange labeling of the nuclei layer in a2 is caused by the high density of nuclei stained with fast nuclear red that does not affect the specific Congo red staining. **(C)** The diagram shows the retinal and cerebral mean number (±SEM) of amyloid positive vessels per animal across all age groups. Each dot represents the data from one investigated animal. For retinal amyloid angiopathy a significant difference between all age groups was found in Kruskal-Wallis test. *post-hoc* pairwise comparisons found significant differences between the groups of 44 weeks and 18/24 weeks as well as between the groups of 32 and 24 weeks for retinal amyloid angiopathy. **(D)** The scatterplot shows the significant correlation between the occurrence of amyloid angiopathy in the retina and in the brain. Each dot represents the data from one investigated animal as the sum of affected vessels in all investigated FOVs. **(E)** The diagram shows the prevalence of retinal and cerebral vascular Aβ accumulations and blood brain/retina barrier breakdown (plasma protein IgG deposits). The brains of all animals (*n* = 24) and the eyes of 11 animals were investigated immunohistochemically. STL, solanum tuberosum lectin (endothelial marker); DAPI, 4'.6-diamidino-2-phenylindole (nuclear staining); Aβ, β-amyloid; IgG, Immunglobulin G (blood brain/retina barrier breakdown detection); *p < 0.05; (a1, a2) Congo red staining, (a3, a4) Thioflavin T staining, **(B)** Immunohistochemistry.

Capillary erythrocyte thrombi were existent in all *n* = 24 (100%), arteriolar erythrocyte thrombi in *n* = 21 (87.5%) animals and both of them could be detected in all brain regions under investigation: 93.3% of the erythrocyte thrombi were found in capillaries and 6.7% in arterioles (Figure 1, subfigures a1,a3). Small perivascular bleeds were existent in *n* = 6 (25.0%) of the animals and were found in the basal ganglia, the hippocampus and the thalamus (Figure 1, subfigures b1,b3). Vascular Aβ deposits (CAA) were existent in *n* = 22 (91.7%) of the rats and were found in all brain regions under consideration (Figure 2, subfigures a1,a3,b1,b3). Table 1 demonstrates the prevalence of rats affected by erythrocyte thrombi, small perivascular bleeds and CAA and the severity of each phenomenon, respectively.

**Table 1:** Cerebral pathologies in the different brain regions.

**Cerebral pathology**	**Brain region**	**Affected animals**	**Mean number**	**Standard deviation**	**Min**	**Max**
Total erythrocyte thrombi	Whole brain	100%/*n* = 24	179.60	303.2	8	1,392
	Cortex	95.8%/*n* = 23	98.00	237.2	0	1,043
	Basal ganglia	91.7%/*n* = 22	22.70	30.5	0	115
	Hippocampus	100%/*n* = 24	32.00	35.7	3	144
	Corpus callosum	70.8%/*n* = 17	9.00	19.9	0	75
	Thalamus	95.8%/*n* = 23	17.40	25.3	0	118
Arteriolar erythrocyte thrombi	Whole brain	87.5%/*n* = 21	12.10	12.8	0	41
	Cortex	58.3%/*n* = 14	3.30	4.1	0	11
	Basal ganglia	62.3%/*n* = 15	2.70	4.1	0	15
	Hippocampus	62.3%/*n* = 15	4.00	5.3	0	17
	Corpus callosum	29.2 %/*n* = 7	0.50	0.9	0	3
	Thalamus	50.0%/*n* = 12	2.10	3.0	0	9
Small perivascular bleeds	Whole brain	25%/*n* = 6	0.29	0.6	0	2
	Cortex	0%/*n* = 0	0.00	0	0	0
	Basal ganglia	4.2%/*n* = 1	0.04	0.2	0	1
	Hippocampus	16.7%/*n* = 4	0.20	0.5	0	2
	Corpus callosum	0%/*n* = 0	0.00	0	0	0
	Thalamus	4.2%/*n* = 1	0.04	0.2	0	1
AA	Whole brain	91.7%/*n* = 22	61.75	83.3	0	259
	Cortex	91.7%/*n* = 22	31.00	43.9	0	143
	Basal ganglia	62.5%/*n* = 15	10.30	16.9	0	50
	Hippocampus	91.7%/*n* = 22	12.80	15.8	0	48
	Corpus callosum	45.8%/*n* = 11	3.50	5.9	0	22
	Thalamus	58.3%/*n* = 14	4.50	6.3	0	20

*The table shows the number of animals affected by erythrocyte thrombi, small perivascular bleeds and cerebral amyloid angiopathy (CAA) in the different brain regions (as percentage and total number of affected animals), related to histological analysis. The mean number of the respective pathology per animal is displayed, including standard deviation, minimum and maximum value (for erythrocyte thrombi the mean number refers to 50 fields of view per brain region, adding up to 250 fields of view for the whole brain; for small perivascular bleeds and CAA means refer to all investigated slices)*.

Within each brain region the vessel density was similar between the three age groups (for detailed numbers see Supplementary Table 1).

The Kruskal-Wallis test showed a significant difference between the age groups for CAA severity [H (4) = 11.2; *p* = 0.024; Figure 2C]. However, *post-hoc* tests did not show any significant results (see Supplementary Table 2). There were no significant differences between the age groups regarding the severity of erythrocyte thrombi (total, capillary, arteriolar) or small perivascular bleeds (Figures 1C,D).

Spearman's correlation displayed a significant positive relationship between age and the whole-brain CAA severity (rho = 0.55, *p* = 0.005). Considering the brain regions separately, the relationship remained significant for the hippocampus and the corpus callosum only (rho = 0.60, *p* = 0.002; rho = 0.64, *p* = 0.001). Age did not relate to the severity of erythrocyte thrombi (total, capillary, arteriolar) or small perivascular bleeds. Absent age effects on erythrocyte thrombi were related to the high number of total and arteriolar erythrocyte thrombi in 18 weeks old SHRSP (Figure 1C) which was not related to an increased vessel density in this age group (see above). All correlation coefficients between age and erythrocyte thrombi, small perivascular bleeds and CAA are given in Supplementary Table 3.

There was no association between the severity of erythrocyte thrombi, small perivascular bleeds and CAA (Supplementary Table 4).

### Retina

Capillary erythrocyte thrombi were existent in *n* = 23 (95.8%) animals, arteriolar erythrocyte thrombi in *n* = 20 (83.8%) animals; 77.2% of the erythrocyte thrombi were found in capillaries and 22.8% in arterioles (Figure 1, subfigures a2,a4). Small perivascular bleeds were found in *n* = 3 (12.5%) of the animals (Figure 1, subfigures b2,b4). All 24 (100%) animals presented vascular Aβ depositions in terms of amyloid angiopathy (AA) (Figure 2, subfigures a2,a4,b2,b4). Table 2 displays the prevalence of SHRSP affected by erythrocyte thrombi, small perivascular bleeds and AA and the severity of each SVD pathology.

**Table 2:** Retinal pathologies.

**Retinal pathology**	**Affected animals**	**Mean number**	**Standard** **deviation**	**Min**	**Max**
Total erythrocyte thrombi	95.8%/*n* = 23	12.5	15.9	0	79
Arteriolar erythrocyte thrombi	83.3%/*n* = 20	3.50	6.6	0	33
Small perivascular bleeds	12.5%/*n* = 3	0.20	0.6	0	2
AA	100%/*n* = 24	26.0	18.1	4	68

*The table shows the number of animals affected by erythrocyte thrombi, small perivascular bleeds, and retinal amyloid angiopathy (AA) (as percentage and total number of affected animals), related to histological analysis. For each pathology the mean number and additionally the standard deviation and minimum and maximum value is displayed (mean number refers to all investigated slices)*.

Kruskal-Wallis test showed a significant difference between the age groups concerning retinal AA severity [H(4) = 19.23; *p* = 0.001; Figure 2C]. Specifically, the group of 44 weeks which showed a significantly higher number of retinal Aβ-positive vessels than the groups of 18 weeks (r = 0.47, *p* = 0.001) and 24 weeks (r = 0.48; *p* = 0.001; Figure 2C). Likewise, the group of 32 weeks showed a significantly higher burden of AA than the group of 24 weeks (r = 0.41, *p* = 0.004; Figure 2C; for other *post-hoc* tests see Supplementary Table 5). Using Spearman's correlation, a significant correlation between age and the number of Aβ-positive vessels could be established (rho = 0.89, *p* < 0.001). For erythrocyte thrombi (total, capillary, arteriolar) and small perivascular bleeds no significant age effect could be detected (Figures 1C,D, Supplementary Table 6).

Severity of total erythrocyte thrombi was related to the severity of retinal small perivascular bleeds (rho = 0.42, *p* = 0.042; not significant after adjustment for multiple comparisons Supplementary Table 7). There was no correlation between the remaining SVD phenomena in the retina (Supplementary Table 7).

### Associations Between Brain and Retina Pathology

Spearman's correlation revealed a significant relationship between the severity of total cerebral and retinal erythrocyte thrombi (rho = 0.46, *p* = 0.023, Figure 1E) as well as between the number of Aβ-positive vessels in the brain and the retina (rho = 0.51, *p* = 0.012; Figure 2D).

Results of ROC curve analysis were as follows: for the prediction of the severity of total cerebral erythrocyte thrombi and the prevalence of cerebral small perivascular bleeds, total retinal erythrocyte thrombi showed the highest Youden-index, while the Youden-index of retinal AA was considerably lower (Table 3). For the prediction of CAA severity, retinal AA displayed the highest Youden-index, while the Youden-index of retinal erythrocyte thrombi was considerably lower (Table 3).

**Table 3:** Prediction of cerebral pathologies by retinal pathologies in spontaneously hypertensive stroke-prone rats.

**Cerebral pathology**	**Threshold** **(weakly vs. strongly affected)**	**Retinal pathology**	**Cutoff value**	**Sensi-tivity**	**Speci-ficity**	**J**	**AUC**
Erythrocyte thrombi	≥81 (*n* = 12 vs. *n* = 12)	Erythrocyte thrombi	6.5	83.3%	66.7%	0.500	0.816
		AA	21	50.0%	41.7	0.083	0.448
Small perivascular bleeds	≥1 (*n* = 18 vs. *n* = 6)	Erythrocyte thrombi	7.5	83.3%	72.2%	0.555	0.546
		AA	17.5	50%	38.9%	−0.111	0.398
CAA	≥30 (*n* = 9 vs. *n* = 15)	Erythrocyte thrombi	6.5	55.6%	40%	−0.044	0.489
		AA	21	88.9%	66.6%	0.556	0.793

This table displays the prediction values for cerebral erythrocyte thrombi, small perivascular bleeds and CAA by retinal arteriolar erythrocyte thrombi, small perivascular bleeds and AA. According to a certain threshold value, all animals were grouped into “weakly” and “strongly” affected by the cerebral pathologies; the thresholds and numbers of weakly and strongly affected animals are given in the table. The following retinal pathologies showed the highest combined sensitivity and specificity correlations for the cerebral pathologies: (i) retinal erythrocyte thrombi for cerebral erythrocyte thrombi, (ii) retinal erythrocyte thrombi for cerebral small perivascular bleeds, and (iii) AA for CAA; the respective values are marked in orange.

*AA, amyloid angiopathy; AUC, area under the curve; CAA, cerebral amyloid angiopathy; J, Youden Index*.

Immunohistochemical analysis revealed cerebral vascular Aβ accumulations in *n* = 11 (45.8%) SHRSP and retinal Aβ deposits in *n* = 7 (63.3%) SHRSP. Cerebral IgG deposits became obvious in *n* = 18 (75.0%) SHRSP and retinal IgG accumulations in *n* = 9 (81.8%) SHRSP. Interestingly, all of the animals, in which vascular Aβ accumulations were existent in either brain or retina, additionally showed perivascular IgG deposits. The prevalence of vascular Aβ deposits and IgG accumulations did not differ between brain and retina (Figure 2E).

## Discussion

Our work on retinal biomarkers for the prediction of CSVD attempted to systematically explore histological associations between cerebral and retinal SVD in SHRSP. This is the first study to detect histological CSVD phenomena such as erythrocyte thrombi, small perivascular bleeds and vascular Aβ accumulations in the retinae of SHRSP. There were medium-effect size correlations between early SVD pathologies (erythrocyte thrombi) and vascular Aβ accumulations in the brain and in the retina. Our results further point toward the potential of retinal SVD markers (erythrocyte thrombi, vascular Aβ accumulations) to predict initial CSVD phenomena (erythrocyte thrombi) and cerebral vascular Aβ deposits. This underlines the important role of the retina as a potential investigative organ for the early diagnosis of CSVD, mutually comprising its subtypes HA (characterized by erythrocyte thrombi) and CAA (characterized by cerebral vascular Aβ deposits). This is of special importance as early HA and CAA are currently not directly measurable *in vivo* in the routine clinical diagnostic setup.

Taken together, our results support the hypothesis that SVD represents a systemic condition. The investigated retinal biomarkers seem to be of good sensitivity for CSVD severity. Severity of retinal erythrocyte thrombi was thereby best to predict the severity of cerebral erythrocyte thrombi, while severity of retinal AA was best in predicting CAA severity. Both pathologies thus seem to develop similarly in both organs. Retinal markers, however, had a comparable low specificity for CSVD with a false positive prediction rate of around 30%. This rate depends on animals with already severe retinal SVD pathologies despite (still) comparably less advanced CSVD. Based on this, one may conclude that retinal SVD precedes CSVD and proceeds faster than CSVD. Even though not significant, higher prevalence of RBB breakdown (IgG deposits) compared to BBB breakdown seem to underline that retinal SVD might have an earlier onset.

There was a lack of CSVD predictability for retinal perivascular microbleeds. This stands in some contrast to one human case series of seven patients with CAA-related intracerebral hemorrhage who all showed multiple retinal dot and blot hemorrhages and several microaneurysms on fundus fluorescein angiography (24). On the other hand, the population-based AGES-Reykjavik study compared eyes and brains of 4,176 individuals and showed that focal arteriolar signs (arteriolar narrowing and arteriovenous nicking), but not retinopathy lesions (blot hemorrhages and microaneurysms), were significantly associated with advanced CSVD downstream pathologies such as WMH and subcortical infarcts (39). This might point to a closer relationship between retinal and cerebral SVD pathologies at more initial disease stages, which seems to be not that strong for advanced SVD pathologies. Strikingly, all of the three animals with retinal microbleeds showed high numbers of cerebral and retinal erythrocyte thrombi, of which the retinal erythrocyte thrombi were able to predict the occurrence of cerebral microbleeds with a high sensitivity, overall pointing toward a similar SVD cascade in the retina and the brain.

Based on our previous research we chose an age of 18 weeks to mirror rather initial CSVD stages (29–31). At the age of around 32–44 weeks most SHRSP provide advanced CSVD stages as well as cerebral parenchymal and vascular Aβ (40). Unexpectedly, the 18 weeks old SHRSP showed the highest number of cerebral capillary and arteriolar erythrocyte thrombi in this sample. These results do not match our former studies (29), in which cerebral erythrocyte thrombi increased with age and peaked at an age of 28–32 weeks (31). However, at least as a trend this age-dependency became obvious in the remaining animals of the current sample aged between 24 and 44 weeks. Retinal erythrocyte thrombi did not increase with age, although their severity nicely correlated with cerebral thrombi of which we know, that number and prevalence rises with age. The prevalence for CAA (92%) was remarkably but was in line with our previous studies in SHRSP (34).

Furthermore, the results confirm the mutual occurrence of HA and CAA not only in the brain but also in the retina, pointing toward the idea of similar underlying risk factors and, possibly, pathophysiological processes. The regional distribution of HA and CAA phenomena in the SHRSP does thereby not necessarily represent the commonly accepted pattern in human of mainly subcortical- (and white matter-) dominant HA and mainly occipital cortical-dominant CAA. Instead, in SHRSP, both, non-amyloid CSVD/HA and CAA seem to spread similarly into cortical and subcortical regions, which might be explained by regional anatomical differences of the vasculature. In humans, the basal ganglia arteriolar perivascular spaces (PVS) communicate directly with the subarachnoid space. Contrary, cortical arteriolar PVS are thought to communicate with the subpial rather than the subarachnoid space (41). These cortical PVS may drain Aβ into the interstitial fluid thus less effectively than in the basal ganglia PVS leading to vascular Aβ accumulations preferably in the walls of cortical vessels. It is reasonable to assume that such regional differences exist in the SHRSP as well, supported by the higher cortical frequency of vascular Aβ deposits, but maybe to a different extent (34, 42–44).

If there is a causal relationship between HA and CAA as well needs to be elucidated. Relationship could be based on cerebral Aβ clearance pathways, which are non-lymphatic (e.g., enzymatic degradation, transport across the BBB) or lymphatic (e.g., drainage along the vessels' basement membranes (intramural periarterial drainage) or along glial water channels of the glymphatic system) (45–47). It was shown that a glymphatic waste clearance system also exists for the retina (48), through which perivascular cleared Aβ retinal can be drained into the meningeal lymphatic vessels, enveloping the optic nerve. Although studies in the early 2000s' mainly focused on non-lymphatic Aβ clearance (49, 50), current research suggests that lymphatic Aβ drainage contributes to a larger portion than expected (51–53). The exact relative contribution of each of these systems to the overall Aβ clearance is currently unknown, but alterations in any of them contribute to extracellular Aβ accumulation (47). HA is supposed to interfere with the normal Aβ clearance processes due to severe vessel wall alterations, potentially facilitating perivascular Aβ accumulations (33, 54). Due to anatomical and physiological similarities in brain and retina these considerations could be true for both organs.

Our study faces several limitation. First, there is a lack of a control group. We can, however, rely on our previous work where age-matched Wistar controls displayed more subtle and early CSVD phenomena (e.g., low numbers of capillary erythrocyte thrombi, occasional BBB disturbances) (29, 31). Advanced CSVD, e.g., perivascular bleeds, occlusive erythrocyte thrombi, vascular Aβ accumulations did not occur in control animals (32, 33, 40, 55). Additionally, Wistar rats are suitable for physiological aging research comprising the retinal vasculature as well: for comparable young age groups as ours several studies demonstrated the absence of a broad spectrum of early retinal (e.g., BRB breakdown, increased inflammation) and more advanced retinal SVD (e.g., local vascular narrowing, reduced capillary density, arteriolar occlusions and microaneurysms) (56–58). Such pathologies became obvious in Wistar rats just at quite old ages of 18–30 months. Thus, we have a quite good knowledge about the isolated cerebral and retinal phenotype of Wistar rats during the investigated age ranges, but admittedly correlation analysis of both organs is still missing. We here presented a new and insightful relationship between pathophysiological processes in the retina and brain of SHRSP, picturing patients with an increased vascular risk profile, and demonstrated a possible model of early cerebral pathology prediction in CSVD, a disease, absent of early, good biomarkers. Definitely, having an animal control group would make our data more valid and reliable, but presenting the results only for SHRSP does not diminish its value here.

Furthermore, we only performed descriptive analysis on single histological and immunohistochemical experiments, with a limited number of variables without expanding our experimental and analytical design, such as including *in vivo* methods or further CSVD and retinal marker. We only counted the number of certain pathologies in a broad analysis of the brain and the retina without further normalizing our results (e.g., to the number of vessels per FOV). But our own unpublished data from previous studies and the investigation of vessel density of exemplary SHRSP from different age groups of our sample proved that small vessel density in SHRSP does not differ between different age groups. Therefore, the absolute numbers from the current study should be comparable between the investigated age groups.

For future studies, we propose a parallel, blinded predictive study design for a further SHRSP sample, in which more markers (e.g., for inflammatory processes, remodeling of the perivascular extracellular matrix, endothelial failure), a bigger sample size and a larger age range should be taken into account, to validate and further develop our results. Of special interest is the relationship of *in vivo* markers such as blood flow measurements in brain and retina (33) which might also be a feasible method for human brains and retinas.

Several *in vivo* human studies show a correlation between changes in the retinal vasculature (e.g., focal arteriolar signs, retinopathy lesions) and CSVD downstream pathology MRI markers (e.g., WMH, subcortical infarcts, hemorrhages) suggesting a close relationship between cerebral and retinal SVD (24, 39). Moreover, in AD patients retinal Aβ plaques were measurable using oral curcumin administration to detect the fluorescence signal with a modified ophthalmoscope (59). Clinical trials further already focus on the assessment of initial CSVD markers, e.g., BBB leakage (assessed through e.g., dynamic contrast enhancement MRI) or blood flow velocity changes (assessed through e.g., velocity phase-contrast MRI) (60–62). In the future, these CSVD measures have to dedicatedly related to *in vivo* retinal biomarkers like wisely indicating initial SVD (e.g., retinal perfusion, capillary/arteriolar blood flow, capillary density (assessed through Optical Coherence Tomography—Angiography (OCTA), BRB failure [assessed through Retinal Leakage Analyzer]) and advanced SVD (e.g., focal arteriolar signs, microbleeds) (63). Cerebral and retinal vascular Aβ assessed through e.g., positron emission tomography, cerebrospinal fluid, and curcumin based retinal fluorescence have to be taken into account as well.

Our present work has to be considered a pilot study on a small postmortem rat sample which just poses a first step for future in-depth research about the brain-retina axis in CSVD. We tried to give special weights to the predictive value of initial retinal SVD markers for CSVD, but future studies should also focus on more advanced vascular pathologies to examine the entire relationship between SVD in different organs and the development of similar cascades.

## Data Availability Statement

The datasets generated for this study are available on request to the corresponding author.

## Ethics Statement

The animal study was reviewed and approved by Animal Care Committee of Saxony-Anhalt (reference numbers: 42502-2-1148 DZNE and 42502-2-1277 Uni/MD).

## Author Contributions

HS, PU, and CG performed all of the experiments and analyzed the data. SS and SJ planned the study and were responsible for supervision and project administration. HS, PU, and SJ visualized the data and wrote the manuscript. HS, PU, CG, RC, H-JH, SS, and SJ reviewed and edited the manuscript and contributed to the interpretation of the data. H-JH was responsible for funding acquisition. All authors contributed to the article and approved the submitted version.

## Conflict of Interest

The authors declare that the research was conducted in the absence of any commercial or financial relationships that could be construed as a potential conflict of interest.
