# Supplementary material for: Retinal Vascular Pathology in a Rat Model of Cerebral Small Vessel Disease
Source: Front Neurol. 2020 Jun 30;11:533. doi: 10.3389/fneur.2020.00533 (PMC7338761; doi:10.3389/fneur.2020.00533)
Supplement: Supplementary file 1 [file Data_Sheet_1.docx]

Supplementary Material

# Supplementary Tables

**Supplementary Table 1: Cerebral vessel density in exemplary SHRSP.**

The table shows the mean number of cerebral capillaries and arterioles per FOV in exemplary animals of three different age groups (18 weeks, 28 weeks, 44 weeks). In total, 15 FOVs of three HE stained brain slices were analyzed for each animal.

| Brain region | Mean number of capillaries / FOV | | | | Mean number of arterioles / FOV | | | |
| --- | --- | --- | --- | --- | --- | --- | --- | --- |
|  | Total  (n = 6) | 18 weeks (n = 2) | 28 weeks (n = 2) | 44 weeks (n = 2) | Total  (n = 6) | 18 weeks (n = 2) | 28 weeks (n = 2) | 44 weeks (n = 2) |
| Cortex | 60.5 | 60.5 | 58.0 | 63.0 | 4.8 | 5.5 | 4.0 | 5.0 |
| Basal ganglia | 47.7 | 51.5 | 46.0 | 45.5 | 3.8 | 2.5 | 4.5 | 3.5 |
| Hippocampus | 47.0 | 49.0 | 45.5 | 46.5 | 3.2 | 2.5 | 3.5 | 3.5 |
| Corpus Callosum | 38.7 | 40.0 | 38.5 | 37.5 | 1.5 | 1.0 | 1.5 | 2.0 |
| Thalamus | 45.5 | 47.5 | 45.0 | 44.0 | 2.2 | 1.5 | 2.5 | 2.5 |

**Supplementary Table 2: Post hoc analysis Kruskal-Wallis test for cerebral amyloidangiopathy among the different age groups.**

The table shows pairwise comparisons of the age groups with corresponding test-statistic (Dunn-Bonferroni test) and p-value. The corrected threshold for multiple testing was
p < 0.005.

| Sample 1 – Sample 2 | Test - Statistic | p-value |
| --- | --- | --- |
| 18 weeks – 24 weeks | -0.63 | 0.900 |
| 18 weeks – 28 weeks | 3.10 | 0.513 |
| 18 weeks – 32 weeks | -8.83 | 0.053 |
| 18 weeks – 44 weeks | -6.40 | 0.177 |
| 24 weeks – 28 weeks | -3.73 | 0.432 |
| 24 weeks – 32 weeks | -8.21 | 0.072 |
| 24 weeks – 44 weeks | -5.78 | 0.223 |
| 28 weeks – 32 weeks | -11.93 | 0.005 |
| 28 weeks – 44 weeks | -9.50 | 0.034 |
| 32 weeks – 44 weeks | 2.43 | 0.570 |

**Supplementary Table 3: Nonparametric correlation analysis for cerebral pathologies and age.**

The table displays the correlation analysis for cerebral erythrocyte thrombi, small perivascular bleeds and cerebral amyloid angiopathy (CAA) and age with Spearman´s rho and the corresponding p-value. The corrected threshold for brain regions was p < 0.01. Significant values are highlighted in orange.

| Cerebral pathology - Age | Spearman´s rho | p-value |
| --- | --- | --- |
| Erythrocyte thrombi (whole brain) – Age | -0.14 | 0.526 |
| Erythrocyte thrombi (cortex) – Age | -0.30 | 0.157 |
| Erythrocyte thrombi (basal ganglia) – Age | -0.04 | 0.870 |
| Erythrocyte thrombi (hippocampus) – Age | -0.36 | 0.087 |
| Erythrocyte thrombi (corpus callosum) – Age | -0.29 | 0.173 |
| Erythrocyte thrombi (thalamus) – Age | -0.22 | 0.304 |
| Small perivascular bleeds (whole brain) – Age | -0.24 | 0.262 |
| Small perivascular bleeds (cortex) – Age | - | - |
| Small perivascular bleeds (basal ganglia) – Age | -0.05 | 0.830 |
| Small perivascular bleeds (hippocampus) – Age | -0.08 | 0.701 |
| Small perivascular bleeds (corpus callosum) – Age | - | - |
| Small perivascular bleeds (thalamus) – Age | -0.31 | 0.144 |
| CAA (whole brain) – Age | 0.55 | 0.005 |
| CAA (cortex) – Age | 0.45 | 0.029 |
| CAA (basal ganglia) – Age | 0.51 | 0.012 |
| CAA (hippocampus) – Age | 0.60 | 0.002 |
| CAA (corpus callosum) – Age | 0.64 | 0.001 |
| CAA (thalamus) – Age | 0.44 | 0.031 |

**Supplementary Table 4: Nonparametric correlation analysis between different cerebral pathologies.**

The table displays the correlation analysis between erythrocyte thrombi, cerebral amyloid angiopathy (CAA) and small perivascular bleeds in the brain with Spearman´s rho and the corresponding p-value. The corrected threshold was p < 0.025.

| Cerebral pathologies | Spearman’s rho | p-value |
| --- | --- | --- |
| Erythrocyte thrombi – CAA | 0.392 | 0.071 |
| Erythrocyte thrombi – small perivascular bleeds | 0.184 | 0.389 |
| CAA – small perivascular bleeds | -0.23 | 0.296 |

**Supplementary Table 5: Post hoc analysis Kruskal-Wallis test for retinal amyloidangiopathy among the different age groups.**

The table shows pairwise comparisons of the age groups with corresponding test-statistic (Dunn-Bonferroni test) and p-value. The corrected threshold for multiple testing was
p < 0.005. Significant values are highlighted in orange.

| Sample 1 – Sample 2 | Test - Statistic | p-value |
| --- | --- | --- |
| 18 weeks – 24 weeks | -3.75 | 0.940 |
| 18 weeks – 28 weeks | -6.75 | 0.154 |
| 18 weeks – 32 weeks | -12.67 | 0.054 |
| 18 weeks – 44 weeks | -15.55 | 0.001 |
| 24 weeks – 28 weeks | -7.13 | 0.132 |
| 24 weeks – 32 weeks | -13.04 | 0.004 |
| 24 weeks – 44 weeks | -15.93 | 0.001 |
| 28 weeks – 32 weeks | -5.92 | 0.166 |
| 28 weeks – 44 weeks | -8.80 | 0.049 |
| 32 weeks – 44 weeks | -2.89 | 0.500 |

**Supplementary Table 6: Nonparametric correlation analysis for retinal amyloid angiopathy (AA) and age.**

The table displays the correlation analysis for retinal AA and age with Spearman´s rho and the corresponding p-value. The corrected threshold was p < 0.025. Significant values are highlighted in orange.

| Retinal pathology - Age | Spearman´s rho | p-value |
| --- | --- | --- |
| Erythrocyte thrombi – Age | -0.36 | 0.083 |
| Small perivascular bleeds – Age | -0.10 | 0.634 |
| AA – Age | 0.89 | < 0.001 |

**Supplementary Table 7: Nonparametric correlation analysis between different retinal pathologies.**

The table displays the correlation analysis between erythrocyte thrombi, small perivascular bleeds and amyloid angiopathy (AA) in the retina with Spearman´s rho and the corresponding p-value. The corrected threshold was p < 0.025.

| Retinal pathologies | Spearman’s rho | p-value |
| --- | --- | --- |
| Erythrocyte thrombi – AA | -0.23 | 0.274 |
| Erythrocyte thrombi – small perivascular bleeds | 0.42 | 0.042 |
| AA – small perivascular bleeds | -0.16 | 0.451 |
